# Digital Self-Efficacy, Satisfaction With the Daily Life Changes Stemming From Digital Transformation, and the Moderating Effect of Social Capital in Middle-Aged and Older Adults: Cross-Sectional Survey Study

**DOI:** 10.2196/79845

**Published:** 2026-07-31

**Authors:** Jeongeun Choi, Hyeonmi Cho, Hyangkyu Lee

**Affiliations:** 1 Mo-Im Kim Nursing Research Institute, College of Nursing and Brain Korea 21 FOUR Project, Yonsei University, Seoul, Republic of Korea; 2 College of Nursing, Research Institute of AI and Nursing Science, Gachon University, Incheon, Gyeonggi-do, Republic of Korea; 3 Mo-Im Kim Nursing Research Institute, College of Nursing, Institute for Innovation in Digital Healthcare, Yonsei University, 50-1 Yonsei-ro, Seodaemun-gu, Seoul, 03722, Republic of Korea, 82 2-2228-3302, 82 2-2227-8303

**Keywords:** digital transformation, self-efficacy, social capital, accessibility, digital integration, digital divide

## Abstract

**Background:**

The swift pace of digital transformation has heightened individuals’ dependence on digital technologies. This makes it imperative to explore how individual factors such as digital self-efficacy and social capital affect satisfaction with the daily life changes stemming from digital transformation. This investigation is particularly essential among middle-aged and older adults because they encounter numerous challenges and opportunities from digital transformation.

**Objective:**

This study examined the moderating effect of social capital on the relationship between digital self-efficacy and satisfaction with the daily life changes stemming from digital transformation among middle-aged and older adults.

**Methods:**

This study used data from the 2022 Digital Divide Survey conducted by the National Information Society Agency in South Korea. The sample included 4155 individuals aged 40 years or older, of whom 71.8% (n=2985) were classified as middle-aged (40‐64 y) and 28.2% (n=1170) as older adults (65‐96 y). Data were analyzed using multiple linear regression models with IBM SPSS (version 27.0) and the PROCESS macro.

**Results:**

Compared with the middle-aged group, the older adult group showed lower levels of digital self-efficacy, social capital, and satisfaction with the daily life changes stemming from digital transformation. The interaction term between digital self-efficacy and social capital was significant in the middle-aged group (B=−0.11, *P*=.002) and the older adult group (B=−0.24, *P*<.001). There was a positive association between digital self-efficacy and satisfaction in both groups, and this association was stronger among those with low social capital. Furthermore, this effect was more pronounced in the older adult group (B=0.71, *P*<.001 for low and B=0.47, *P*<.001 for high social capital) than in the middle-aged group (B=0.55, *P*<.001 for low and B=0.46, *P*<.001 for high social capital). Education level and digital competence were positively associated with satisfaction in both age groups; higher household income was positively associated with satisfaction only in the middle-aged group.

**Conclusions:**

Individuals with low social capital showed a stronger association between digital self-efficacy and satisfaction with the daily life changes stemming from digital transformation. Enhancing digital self-efficacy among older adults, especially those with low levels of social capital, may improve their satisfaction with the daily life changes stemming from digital transformation.

## Introduction

### Background

The COVID-19 pandemic expedited digital transformation, increasing the adoption of digital technology-based public and private services and accelerating innovation in information and communication technologies (ICTs) [1,2]. As of 2023, approximately 5.16 billion people (constituting 64.4% of the global population) were using the internet, and approximately 5.44 billion (or 68% of the global population) were using mobile phones [3]. South Korea is at the forefront of this global trend. According to the Organisation for Economic Co-operation and Development (OECD) Digital Government Index published in 2025, South Korea ranked first overall, with a composite score of 0.94, substantially higher than the OECD average of 0.61 [4]. This reflects an environment in which digital platforms are no longer optional but function as essential infrastructure for daily life. This pervasive digitalization has fostered the development of a well-connected society in South Korea. However, this rapid digitalization coincides with an unprecedented pace of population aging.

The global proportion of individuals aged 65 years and older is projected to rise to 1 in 6 by 2050 [5]. Notably, South Korea is experiencing the world’s most rapid pace of population aging and is expected to become one of the world’s most aged societies by 2050, with older adults comprising approximately 40% of the total population [5]. In this context, where rapid digital transformation and population aging are occurring simultaneously, the digital divide extends beyond access to resources to include disparities in digital skills and perceptions of digital technologies [6].

The digital divide refers to the disparity between individuals with access to modern ICTs and those without such access [7]. It contributes to digital exclusion [2], particularly in societies where technology and the internet govern various aspects of daily activities [8,9]. This technological shift brings about changes in daily life that individuals perceive as either beneficial or burdensome. When individuals are digitally excluded, barriers to accessing information and services, as well as maintaining social participation, can undermine everyday autonomy, thereby reducing satisfaction with the daily life changes stemming from digital transformation [9].

While internet adoption has accelerated across all ages in recent years, middle-aged and older adults continue to account for a large proportion of those experiencing the digital divide [10,11]. This population is often less familiar with digital technologies, which makes them more vulnerable to the digital divide and increases their risk of digital exclusion [2,8-12]. Given that digital exclusion can detrimentally affect the quality of life and social participation [13,14], it is essential to address digital divide concerns among middle-aged and older adults. However, the digital divide is not a uniform phenomenon, as its underlying mechanisms and consequences often vary across life stages. From a life-course perspective, middle-aged adults often face strong demands to use digital technologies for work, family responsibilities, and social roles, whereas older adults may encounter more complex barriers due to retirement-related transitions, changes in social ties, and health or functional limitations [15,16].

Previous research suggests that digital technology adoption processes differ between middle-aged and older adults, particularly in how key adaptive resources such as digital self-efficacy and social capital are developed and used. Middle-aged adults, who have higher levels of participation in the workforce, tend to strengthen their digital self-efficacy through task-related experiences, training, and repeated ICT use in both occupational and everyday contexts [17]. In contrast, older adults are more likely to encounter digital technologies later in life and tend to rely more on relational support, such as encouragement, demonstration, and guidance from family members or social networks during the adaptation process [18]. The use of social capital in digital environments also differs across age groups. Middle-aged adults often use broader and more diverse networks, including professional and extended social ties, to access information and resources. In contrast, older adults tend to depend on close ties, such as family members and familiar contacts, for instrumental assistance and emotional support [19,20]. Taken together, structural differences in the formation of digital self-efficacy and the usage of social capital between middle-aged and older adults support distinguishing these groups to capture heterogeneity in digital adaptation processes across life stages.

The impact of digital transformation on daily life extends beyond technology use and reflects individuals’ experiences of changes in the digital environment. Recent studies have shown that digital transformation is associated with a range of subjective life outcomes, including life satisfaction [21], quality of life [22], subjective well-being [23], and overall well-being [24]. These indicators capture general life outcomes and assess the effects of digital transformation only indirectly, without reflecting individuals’ evaluations of specific changes in their daily lives. In this study, the dependent variable was measured as “satisfaction with the daily life changes stemming from digital transformation.” This variable reflects how individuals evaluate the impact of accelerated digital changes across key domains of everyday life, including leisure opportunities, access to information and knowledge, social connection and exchange, and work and study efficiency.

This conceptualization is consistent with the third-level digital divide perspective, which emphasizes that digital inequalities are not limited to differences in internet access (the first-level digital divide) and digital skills (the second-level digital divide) [25], and are ultimately reflected in differences in the tangible benefits individuals derive from using technologies [26]. Previous studies further demonstrate that digital transformation is closely associated with key domains of everyday life. Digital transformation facilitates access to information and knowledge by enhancing opportunities for learning and information seeking [27], promotes social connection and exchange by reducing social isolation and supporting social well-being [14,28], and contributes to improved learning experiences and work-related productivity in educational and occupational contexts [29]. Furthermore, digital transformation has extended into leisure, reshaping recreational activities and influencing individuals’ daily experiences [30,31]. Collectively, the dependent variable captures a theoretically grounded measure of perceived satisfaction with digital transformation–related changes in everyday life as a key outcome of digital engagement.

Digital self-efficacy refers to an individual’s belief in their capability to use digital systems effectively [32]. It promotes the long-term use of digital devices, thereby reducing the digital divide and boosting the quality of life [33-35]. According to the self-efficacy theory, self-efficacy is one’s belief in their ability to plan and successfully perform the activities needed to achieve a specific goal [36]. Self-efficacy acts as a resource in task attainment, as individuals regulate their effort and perseverance based on their self-efficacy [37]. Insufficient digital self-efficacy can hamper the ability of middle-aged adults and older adults to effectively use technologies to address later-life needs, such as financial management, health care, and retirement planning [8,38]. Addressing the barriers to technology use and enhancing digital self-efficacy may improve their engagement and proficiency with digital devices, which can, in turn, aid them in achieving successful aging [33,39].

Social capital is defined as networks and relationships among individuals that are underpinned by the norms of reciprocity and trust. It encompasses two key functions: (1) bonding, which creates strong emotional connections; and (2) bridging, which produces weaker but broader connections [40]. As individuals transition from middle age to older adulthood, events such as retirement, the loss of spouses and close connections, limited educational opportunities, and mobility limitations diminish social capital. Specifically, there is a gradual reduction in one’s social networks and the capacity to use social resources [41-43]. Social capital is a significant contributor to the use of digital devices and the maintenance of online engagement [38]. Additionally, it can function as a contextual resource that moderates the relationship between digital self-efficacy and satisfaction with the daily life changes stemming from digital transformation [44]. When social capital is high, individuals can access support that facilitates digital engagement and reduces reliance on their own digital self-efficacy, whereas when social capital is low, limited support increases dependence on digital self-efficacy for digital engagement [45]. Accordingly, the effect of digital self-efficacy on satisfaction with the daily life changes stemming from digital transformation may vary depending on the level of social capital.

According to the conservation of resources (COR) theory [46], individuals experiencing resource loss tend to protect and preserve their remaining resources. In technology-dependent societies, individuals with low social capital have limited access to external support, increasing their vulnerability to resource loss. In this context, digital self-efficacy may function as a key internal resource that compensates for the lack of external support. Specifically, higher digital self-efficacy may enable individuals to more effectively navigate digital environments despite constrained support, thereby facilitating a more adaptive appraisal of digital transformation as manageable rather than threatening [47]. Such appraisal may reduce maladaptive responses and enhance satisfaction with the daily life changes stemming from digital transformation. Taken together, these considerations suggest that digital self-efficacy may serve as a compensatory resource among middle-aged and older adults with limited social capital, with important implications for supporting adaptation to evolving digital environments and reducing the digital divide.

### Purpose

This study aimed to evaluate the relationship between digital self-efficacy, social capital, and satisfaction with the daily life changes stemming from digital transformation. Specifically, it focused on how social capital moderates the relationship between digital self-efficacy and satisfaction with the daily life changes stemming from digital transformation among both middle-aged and older adults.

## Methods

### Data and Study Population

We used cross-sectional data from the 2022 Digital Divide Survey, an annual nationwide survey conducted by the Ministry of Science and ICT and the National Information Society Agency in South Korea. In this study, analyses were based on the general population subsample aged 40 years or older (n=4155). Adults aged 40‐64 years were classified as the middle-aged group (n=2985, 71.8%), and those aged 65‐96 years as the older adult group (n=1170, 28.2%), based on the official life-stage age classification used in Korea’s national administrative statistics [48]. In 2022, the survey was conducted from September to December using standardized face-to-face interviews and used a stratified probability-proportional-to-size sampling design to ensure national representativeness. The survey assessed the effectiveness of policies aimed at reducing the digital divide and included a total of 15,000 respondents from the general population, along with additional samples from vulnerable groups such as older adults, individuals with disabilities, individuals in low-income households, immigrant women in international marriages, North Korean defectors, and individuals engaged in agriculture or fishing. The official methodology report of this survey did not provide an overall response rate; however, the analytic dataset contained no missing values for study variables.

### Variables

#### Dependent Variable

Satisfaction with the daily life changes stemming from digital transformation was determined using 4 items rated on a 5-point Likert scale from 1 (“strongly disagree”) to 5 (“strongly agree”). The items were as follows: (1) “The digital transformation has increased opportunities for leisure activities and made them more enjoyable (for instance, through online content and mobile applications)”; (2) “The digital transformation has enabled quicker access to new information or knowledge (for instance, through remote learning and online content platforms)”; (3) “The digital transformation has increased opportunities to engage with new people and exchange opinions”; and (4) “Efficiency in work or academic pursuits has improved due to practices like remote work and mobile learning.” The total score was calculated as the mean of 4 items, with higher scores indicating greater satisfaction levels [49]. In this study, the scale exhibited excellent internal consistency, with a Cronbach α of 0.90.

Construct validity was examined using confirmatory factor analysis (CFA) in the total sample and separately in middle-aged and older adults, given that this 4-item measure was newly added to the 2022 survey. Model fit was evaluated using the comparative fit index (CFI), Tucker-Lewis index (TLI), and standardized root-mean-square residual (SRMR). The chi-square statistic was excluded from model fit evaluation because it is overly sensitive to large sample sizes [50]. Acceptable model fit was indicated by CFI and TLI≥0.90 and SRMR<0.08 [51], with more stringent cutoffs of CFI and TLI≥0.95 suggested [52]. In addition to CFI, TLI, and SRMR, root-mean-square error of approximation (RMSEA)<0.08 is commonly used as a criterion for acceptable model fit; however, RMSEA tends to be upwardly biased in models with small dfs [53]. Since our measurement model for the 4-item scale has a small df (df=2), model fit was primarily evaluated using CFI, TLI, and SRMR, which are more robust in such cases [53]. Construct reliability was assessed using composite reliability (CR; ≥0.70) [54], and convergent validity was assessed using average variance extracted (AVE; ≥0.50) [55].

#### Independent Variable

Digital self-efficacy was assessed using 4 items rated on a 4-point scale ranging from 1 (“strongly disagree”) to 4 (“strongly agree”). These 4 items have been widely used in past studies [56-58]. The items are as follows: (1) “I am confident that I can learn about digital devices”; (2) “I am confident in using digital devices”; (3) “I can quickly learn how to use new digital devices”; and (4) “I want to use digital devices more frequently.” The digital self-efficacy score was calculated based on the average score on the 4 items, with higher scores reflecting higher digital self-efficacy. In this study, the scale demonstrated excellent internal consistency, with a Cronbach α of 0.88.

#### Moderating Variable

Social capital was measured with an abbreviated version of Williams’s social-capital scale [59] developed by the agency that conducted the 2022 Digital Divide Survey. It comprises 10 items—5 items each for bonding and bridging social capital. The items for bonding social capital were (1) “I know someone who can help me solve my problems”; (2) “I know someone who I can ask for advice when I have to make important decisions”; (3) “I know someone who I can talk to about my private matters”; (4) “I know someone who I can trust with important work”; and (5) “I know someone who can help me fight injustice.” The items for bridging social capital were (1) “I feel connected to a bigger world as I interact with people online and offline.”; (2) “I feel that everyone in the world is connected as I interact with people online and offline.”; (3) “I am willing to spend time on community activities”; (4) “Online and offline interactions allow me to communicate with new people”; and (5) “Online and offline interactions allow me to meet new people.” The items are rated on a 4-point scale that ranges from 1 (“strongly disagree”) to 4 (“strongly agree”). We used a combined social capital score (bonding plus bridging) to capture overall accessible social resources, consistent with previous research [44,58,60]. This operationalization aligned with our focus on moderation by overall social support and information pathways. The average of the scores on the 10 items indicates the total social capital score. Higher scores indicate greater social capital. This scale has been extensively adopted in previous studies [44,58,60]. In this study, the scale had a Cronbach α of 0.84, indicating great internal consistency.

#### Covariates

The general characteristics of the study population and digital competence were included as covariates. The general characteristics were age, sex, education level, living arrangements, the presence of disability, monthly household income, self-rated health, and digital competence. These covariates were selected based on previous studies [32,61,62]. The exchange rate in 2022 was KRW 1=US $0.000774, based on the average annual exchange rate reported by the World Bank [63]. Self-rated health was captured with a single question on how satisfied participants felt about their physical and mental health, with responses ranging from 1 (“not satisfied at all”) to 4 (“very satisfied”) [64]. Digital competence was measured with 14 items assessing personal computer and mobile phone use skills, each rated from 1 to 4, where higher scores indicated greater digital competence [65]. In this study, the scale exhibited a Cronbach α of 0.96, suggesting excellent internal consistency.

### Ethical Considerations

This was a secondary analysis study that used publicly available data without any personally identifiable information. Therefore, this study was exempt from review by the Institutional Review Board (IRB 4-2023-1516).

### Statistical Analysis

We compared the general characteristics of the study population and the study variables between middle-aged and older adults using chi-square tests and 2-tailed independent *t* tests. To examine the associations between digital self-efficacy, social capital, and satisfaction with the daily life changes stemming from digital transformation, we used multiple linear regression analyses. Three separate regression models were developed for the total sample, for middle-aged adults, and for older adults. All regression models were adjusted for covariates. The continuous variables were mean-centered before the regression models were constructed, and no variables were standardized. The moderation effect of social capital was assessed using an interaction term (digital self-efficacy×social capital) in the models. The PROCESS macro in IBM SPSS Statistics was used to determine significant interaction effects. Unstandardized simple slopes were calculated at 1 SD above (+1 SD) and below (−1 SD) the mean score for social capital.

Considering the potential heterogeneity across the dependent variable’s subdimensions, we performed an additional analysis using a multiple indicators multiple causes (MIMIC) model, a specific form of structural equation modeling (SEM) [66]. In this model, the dependent variable was specified as a latent factor indicated by 4 reflective items, while observed covariates were included as causal predictors [67].

Statistical analyses were performed using SPSS Statistics for Windows (version 27.0). The CFA and SEM models were estimated in R (version 4.5.1; R Core Team) using the *lavaan* package, using robust maximum likelihood estimation [68]. We evaluated normality by inspecting absolute skewness values for all continuous variables (with values ≥3 indicating substantial deviation from normality) and visually reviewing histograms and quantile-quantile (Q-Q) plots. All absolute skewness values were <1.5, and no marked deviations from normality were observed in the histograms or Q-Q plots. No missing values were present for any variables included in the analytic sample; therefore, no exclusions or imputations were required. No multicollinearity issues were observed in any of the models because the variance inflation factor remained under 10.

## Results

### General Characteristics of the Study Population

The average age of middle-aged adults was 52.42 (SD 6.80) years, while that of older adults was 71.33 (SD 5.05) years (Table 1). The proportion of individuals with high school or higher educational attainment was higher in the middle-aged group (2814/2985, 94.3%) than that in the older adult group (510/1170, 43.6%). The proportion of individuals living with others was lower in the older adult group (927/1170, 79.2%) than that in the middle-aged group (2786/2985, 93.3%). Furthermore, older adults had lower household incomes, poorer self-rated health, and weaker digital competence than middle-aged adults.

**Table 1.** General characteristics of the study population (N=4155).

Variable	Total (n=4155)	Middle-aged adults (n=2985, 71.8%)	Older adults (n=1170, 28.2%)	*t* test (*df*) or chi-square test (*df*)	*P* value
Age (in y), mean (SD)	57.74 (10.62)	52.42 (6.80)	71.33 (5.05)	−97.93^a^ (4,153)	<.001
Sex, n (%)					
Male	2054 (49.4)	1502 (50.3)	552 (47.2)	3.31^b^ (1)	.07
Female	2101 (50.6)	1483 (49.7)	618 (52.8)		
Education level, n (%)					
Middle school or lower	831 (20)	171 (5.7)	660 (56.4)	1349.40^b^ (1)	<.001
High school or above	3324 (80)	2814 (94.3)	510 (43.6)		
Living arrangements, n (%)					
Living alone	442 (10.6)	199 (6.7)	243 (20.8)	175.85^b^ (1)	<.001
Living with others	3713 (89.4)	2786 (93.3)	927 (79.2)		
Presence of disability, n (%)					
No	4055 (97.6)	2911 (97.5)	1144 (97.8)	0.24^b^ (1)	.63
Yes	100 (2.4)	74 (2.5)	26 (2.2)		
Monthly household income (KRW), n (%)^c^					
<4,000,000	1912 (46)	949 (31.8)	963 (82.3)	863.44^b^ (1)	<.001
≥4,000,000	2243 (54)	2036 (68.2)	207 (17.7)		
Self-rated health, mean (SD)	2.76 (0.66)	2.88 (0.61)	2.47 (0.69)	17.53^a^ (4,153)	<.001
Digital competence, mean (SD)	4.80 (1.65)	5.36 (1.41)	3.37 (1.33)	41.39^a^ (4,153)	<.001
Digital self-efficacy, mean (SD)	2.46 (0.72)	2.67 (0.62)	1.93 (0.68)	32.13^a^ (4,153)	<.001
Social capital, mean (SD)	2.82 (0.45)	2.89 (0.41)	2.65 (0.50)	14.76^a^ (4,153)	<.001
Satisfaction with the daily life changes stemming from digital transformation, mean (SD)	3.01 (0.87)	3.23 (0.75)	2.44 (0.88)	27.17^a^ (4,153)	<.001

a tested using independent *t* test.

b tested using the chi-square test.

c A currency exchange rate of KRW 4,000,000=US $3096.81 (KRW 1=US $0.000774; 2022 annual average, World Bank) was applied [63].

### Differences in Digital Self-Efficacy, Social Capital, and Satisfaction With the Daily Life Changes Stemming From Digital Transformation Between Middle-Aged and Older Adults

The older adult group (mean 1.93, SD 0.68) exhibited lower levels of digital self-efficacy than the middle-aged group (mean 2.67, SD 0.62; *t*_4,153_=32.13; *P*<.001; Table 1). The former (mean 2.65, SD 0.50) demonstrated lower levels of social capital than the latter (mean 2.89, SD 0.41; *t*_4,153_=14.76; *P*<.001). The former (mean 2.44, SD 0.88) also reported lower levels of satisfaction with the daily life changes stemming from digital transformation than the latter (mean 3.23, SD 0.75; *t*_4,153_=27.17; *P*<.001).

### CFA Results for the 4-Item Measure of the Dependent Variable

CFA supported a unidimensional structure of the 4-item measure of satisfaction with the daily life changes stemming from digital transformation in the total sample and both age groups (Table 2). Standardized factor loadings were high (total: 0.82‐0.86; middle-aged: 0.75‐0.82; and older adults: 0.84‐0.88), and fit indices indicated acceptable to excellent fit overall (CFI and TLI≥0.95; SRMR<0.08). Construct reliability and convergent validity were well-supported across groups (CR≥0.7; AVE≥0.5).

**Table 2.** Confirmatory factor analysis results for the dependent variable (N=4155).

Sample and item	Factor loading		Goodness-of-fit indicators				
	β^a^	*P* value	CFI^b^	TLI^c^	SRMR^d^	CR^e^	AVE^f^
Total (n=4155)			0.99	0.99	0.01	0.9	0.7
Item 1: Leisure opportunities	0.83	<.001					
Item 2: Access to information and knowledge	0.82	<.001					
Item 3: Social connection and exchange	0.85	<.001					
Item 4: Work and study efficiency	0.86	<.001					
Middle-aged adults (n=2985)			0.99	0.98	0.01	0.87	0.62
Item 1: Leisure opportunities	0.77	<.001					
Item 2: Access to information and knowledge	0.75	<.001					
Item 3: Social connection and exchange	0.80	<.001					
Item 4: Work and study efficiency	0.82	<.001					
Older adults (n=1170)			0.99	0.98	0.01	0.92	0.73
Item 1: Leisure opportunities	0.84	<.001					
Item 2: Access to information and knowledge	0.84	<.001					
Item 3: Social connection and exchange	0.88	<.001					
Item 4: Work and study efficiency	0.87	<.001					

a β: standardized regression coefficient.

b CFI: comparative fit index.

c TLI: Tucker-Lewis index.

d SRMR: standardized root-mean-square residual.

e CR: composite reliability.

f AVE: average variance extracted.

### Associations Between Digital Self-Efficacy, Social Capital, and Satisfaction With the Daily Life Changes Stemming From Digital Transformation

Higher levels of digital self-efficacy were associated with higher levels of satisfaction with the daily life changes stemming from digital transformation in the middle-aged group (B=0.50, *P*<.001) and in the older adult group (B=0.59, *P*<.001; Table 3). Similarly, higher social capital was significantly associated with higher satisfaction with the daily life changes stemming from digital transformation among both middle-aged (B=0.28, *P*<.001) and older adults (B=0.12, *P*=.01).

**Table 3.** Associations between digital self-efficacy, social capital, and satisfaction with the daily life changes stemming from digital transformation (N=4155).

Variable^a^	Model 1 (total; N=4155)			Model 2 (middle-aged adults; n=2985, 71.8%)			Model 3 (older adults; n=1170, 28.2%)		
	B^b^ (SE)	*t* test (*df*)	*P* value	B (SE)	*t* test *(df*)	*P* value	B (SE)	*t* test *(df*)	*P* value
Independent variable									
Digital self-efficacy	0.53 (0.02)	27.87 (4,143)	<.001	0.50 (0.02)	22.59 (2,973)	<.001	0.59 (0.04)	16.15 (1,158)	<.001
Moderation variable									
Social capital	0.22 (0.03)	8.44 (4,143)	<.001	0.28 (0.03)	9.08 (2,973)	<.001	0.12 (0.05)	2.60 (1,158)	.01
Interaction term									
Digital self-efficacy×social capital^c^	−0.11 (0.03)	−4.01 (4,143)	<.001	−0.11 (0.04)	−3.13 (2,973)	.002	−0.24 (0.06)	−4.05 (1,158)	<.001
Control variables^d^									
Age	−0.003 (0.001)	−2.22 (4,143)	.03	−0.002 (0.002)	−0.91 (2,973)	.36	−0.01 (0.01)	−1.65 (1,158)	.10
Sex (ref^e^: male)	0.02 (0.02)	1.04 (4,143)	.30	0.02 (0.02)	1.00 (2,973)	.32	−0.01 (0.04)	−0.32 (1,158)	.75
Education level (ref: middle school or lower)	0.14 (0.03)	4.32 (4,143)	<.001	0.12 (0.05)	2.43 (2,973)	.02	0.09 (0.05)	1.99 (1,158)	.047
Living arrangements (ref: living alone)	0.002 (0.03)	0.06 (4,143)	.96	−0.003 (0.05)	−0.07 (2,973)	.95	−0.04 (0.05)	−0.82 (1,158)	.41
Presence of disability (ref: without disability)	−0.01 (0.06)	−0.21 (4,143)	.83	−0.09 (0.07)	−1.23 (2,973)	.22	0.16 (0.14)	1.16 (1,158)	.25
Monthly household income (ref: <KRW 4,000,000)^f^	0.09 (0.02)	3.66 (4,143)	<.001	0.11 (0.03)	4.12 (2,973)	<.001	0.02 (0.06)	0.37 (1,158)	.71
Self-rated health	−0.03 (0.02)	−1.58 (4,143)	.12	−0.03 (0.02)	−1.56 (2,973)	.12	−0.03 (0.03)	−0.90 (1,158)	.37
Digital competence	0.08 (0.01)	9.40 (4,143)	<.001	0.07 (0.01)	6.51 (2,973)	<.001	0.13 (0.02)	7.02 (1,158)	<.001

a All continuous predictors were mean-centered prior to creating the interaction term.

b B: unstandardized regression coefficients.

c Conditional effects (simple slopes) of digital self-efficacy were probed at low (−1 SD) and high (+1 SD) levels of social capital and are presented in Figure 1.

d All models were adjusted for the same set of covariates (age, sex, education level, living arrangements, disability, household income, self-rated health, and digital competence).

e ref: reference category.

f A currency exchange rate of KRW 4,000,000=US $3096.81 (KRW 1=US $0.000774; 2022 annual average, World Bank) was applied [63].

### Moderating Role of Social Capital

The interaction effect of digital self-efficacy and social capital was significant among both middle-aged (B=−0.11, *P*=.002) and older adults (B=−0.24, *P*<.001; Table 3). We estimated the unstandardized simple slopes of these relationships (Figure 1). High and low levels of social capital were determined using SD, and values 1 SD above and 1 SD below the mean indicated high and low levels of social capital, respectively. In both the middle-aged and older adult groups, digital self-efficacy displayed a stronger positive relationship with satisfaction with the daily life changes stemming from digital transformation (satisfaction) when social capital was low. Additionally, this relationship was stronger in the older adult group (B=0.71, *P*<.001 for low and B=0.47, *P*<.001 for high social capital) than in the middle-aged group (B=0.55, *P*<.001 for low and B=0.46, *P*<.001 for high social capital). Among covariates, education level and digital competence were positively associated with satisfaction in all groups; household income was positively associated with satisfaction only in the total sample and middle-aged adults, and age was negatively associated with satisfaction only in the total sample.

These results were largely consistent in additional SEM analyses using a MIMIC model (Multimedia Appendices 1-3), particularly with respect to the effects of digital self-efficacy and the digital self-efficacy×social capital interaction term across groups. The direct effect of social capital was significant in the total and middle-aged samples, but was attenuated and not statistically significant among older adults in SEM.

To further examine age heterogeneity, we conducted age-stratified analyses (40‐50 y: n=1189, 51‐64 y: n=1796, 65‐74 y: n=872, and ≥75 y: n=298; Multimedia Appendix 4). In the 65‐74 years group, digital self-efficacy was positively associated with satisfaction with the daily life changes stemming from digital transformation (B=0.60, *P*<.001), and the interaction effect was negative and statistically significant (B=−0.27, *P*<.001). In the ≥75 years group, the interaction term did not reach statistical significance (B=−0.21, *P*=.18), whereas the direct effect of digital self-efficacy remained significant (B=0.54, *P*<.001).

**Figure 1.**
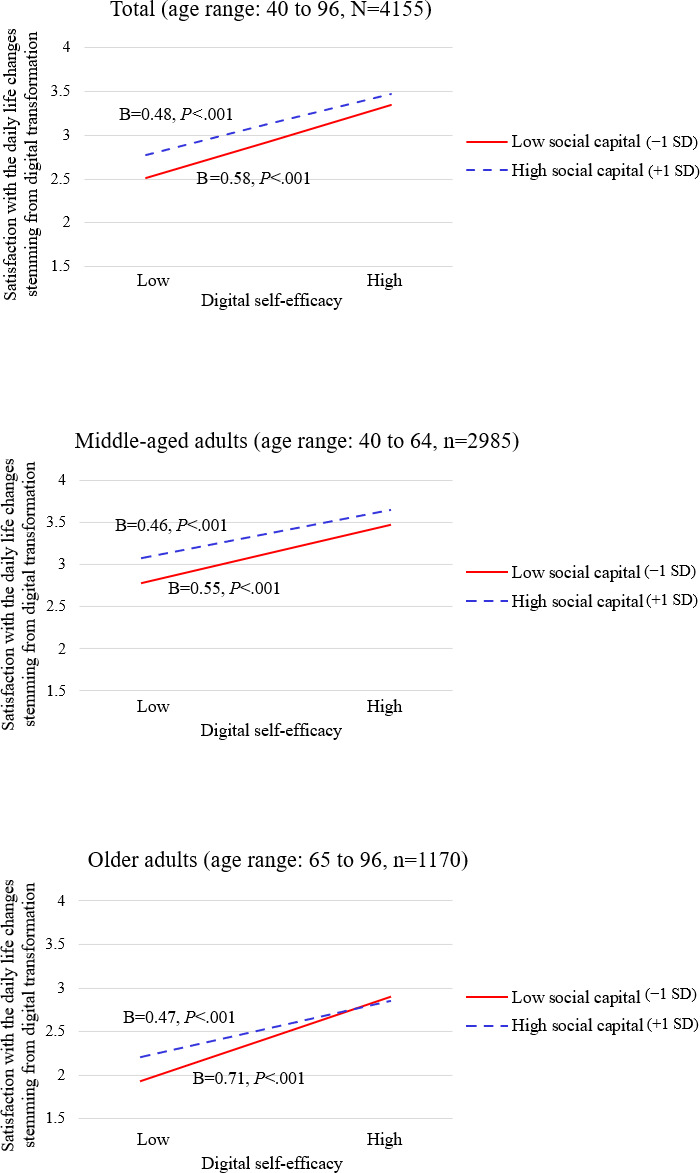
Graphical representation of the moderation effect of social capital in the relationship between digital self-efficacy and satisfaction with the daily life changes stemming from digital transformation.

## Discussion

### Principal Findings

This study examined the moderating effect of social capital on the relationship between digital self-efficacy and satisfaction with the daily life changes stemming from digital transformation among middle-aged and older adults. In both groups, digital self-efficacy was positively linked to satisfaction, and the effect was stronger among individuals with low social capital compared with those with high social capital. Notably, this positive relationship was more pronounced among older adults than among middle-aged adults. These results offer valuable insights into the challenges of digital transformation in aging populations. Importantly, satisfaction with the daily life changes stemming from digital transformation remains underexamined compared with broader outcomes such as quality of life. Focusing on this transformation-specific outcome helps capture individuals’ subjective adaptation to rapidly changing digital environments, which may not be fully reflected in broader well-being indicators. This study extends previous research by demonstrating that social capital moderates the association between digital self-efficacy and satisfaction with the daily life changes stemming from digital transformation, with clear differences across age groups.

Our findings revealed that for individuals with low social capital, digital self-efficacy was more strongly associated with satisfaction with the daily life changes stemming from digital transformation. This was particularly evident among older adults, who may face greater challenges in adapting to digital advancements. These findings suggest that older adults with limited social connections may rely more heavily on their own digital self-efficacy to maintain social engagement, access services, and maintain satisfaction with the daily life changes stemming from digital transformation. Enhancing digital self-efficacy may support more positive perceptions of digital transformation, particularly among socially vulnerable groups, consistent with the COR theory [46]. Similarly, past studies have shown that the positive effect of frequent internet use on life satisfaction is stronger among older adults living alone compared with those living with others [61]. Likewise, the autonomy benefits of active internet use have been reported to be greater among older adults with poorer functional health than among those with better functional health [69].

The moderation effect of social capital reached statistical significance among adults aged 65‐74 years but not among those aged ≥75 years. However, among the old-old (≥75 y; n=298; B=−0.21; *P*=.18), the coefficient was comparable in direction and magnitude to that observed in the young-old (65‐74 y; n=872; B=−0.27; *P*<.001). This suggests that the nonsignificant finding in the old-old group may reflect limited statistical power rather than the absence of a meaningful moderating pattern. Although the directional pattern was broadly consistent across older adult subgroups, these age-stratified findings may also indicate heterogeneity within the older adult population. The young-old (65‐74 y) often experience major life transitions such as retirement, accompanied by shifts in social roles and available resources; thus, digital self-efficacy may function more prominently as a compensatory resource [70]. In contrast, among adults aged ≥75 years, satisfaction with the daily life changes stemming from digital transformation may be shaped not only by digital self-efficacy and social capital but also by structural and functional constraints, such as physical limitations, cognitive burden, poorer health, and accessibility barriers [20,71].

The observed age-related differences may also be interpreted in light of a potential digital cohort effect, whereby digital adaptation is shaped more by the timing and context of initial exposure to digital technologies than by chronological age alone [25,72]. Earlier and sustained exposure to digital technologies may strengthen digital self-efficacy and its influence on satisfaction, whereas later or limited exposure may constrain digital skills and self-efficacy, making it more difficult to effectively use social capital in digital contexts. This interpretation is consistent with previous research emphasizing the role of experience and cumulative engagement with digital technologies in shaping the digital divide among older adults [20]. Although the moderation effect was not statistically significant in the old-old group, digital self-efficacy remained significantly associated with satisfaction. This suggests that digital self-efficacy still plays an important role in this group, consistent with previous findings showing that it continues to influence behavioral intention to use the internet in old-old age [73].

Our results highlight the importance of self-efficacy, as also suggested in social cognitive theory [36]. Self-efficacy is particularly relevant in later life, as this stage is characterized by resource losses [74]. Strategies such as persuasion, observational learning, and mastery experiences can effectively enhance self-efficacy, enabling individuals to balance their actions and acquire new skills [36]. Notably, previous research has consistently identified digital self-efficacy as a key factor in digital proficiency, learning outcomes, and life satisfaction [56,74,75]. Therefore, improving digital self-efficacy may foster acceptance of digital services and confidence in navigating them, supporting greater satisfaction with the daily life changes stemming from digital transformation.

Building upon theoretical foundations, our findings suggest that policy and program designs can benefit from differentiated strategies for middle-aged and older adults. For middle-aged adults, who are often required to engage with digital technologies in occupational and administrative contexts, interventions should focus on enhancing the efficient and context-specific use of digital services. Program impact may be strengthened by long-term outreach that enhances exposure and awareness, alongside a shift toward skill-building-based empowerment and low-barrier service pathways.

For older adults, particularly those with low social capital, interventions could prioritize strengthening digital self-efficacy through tailored support, including dedicated one-on-one mentorship to provide guided mastery experiences. Such support can emphasize confidence-building, achievable goal setting, and the development of organizational and self-management skills. In addition, heterogeneity within older adults should be considered. The young-old may be more likely than the old-old to have previous experience with digital technologies before retirement, and such experience has been shown to be a strong predictor of internet use in later life [20]. Previous experience may facilitate continued learning and make the young-old more responsive to structured, skill-building support such as one-on-one mentorship, enabling the translation of existing familiarity into effective skills and rapid mastery. Therefore, interventions for the young-old may benefit from focusing on such skill-oriented, experience-based support. In contrast, interventions for the old-old should prioritize reducing barriers to access and use, such as simplifying interfaces and providing assistive support. They may also benefit from approaches that enhance digital self-efficacy through personalized, family-integrated support, with attention to accessibility, usability, and functional limitations, including visual and hearing impairments [20,71]. In South Korea, government initiatives to promote digital literacy have included kiosk usage training at senior welfare centers, along with efforts to transform over 2000 senior centers into smart facilities by 2024 [76]. Nonetheless, older adults continue to report low digital self-efficacy and limited awareness of online government services [77], indicating that further expansion and active dissemination are needed to enhance program reach and impact. These efforts may further prioritize subgroups facing compounded barriers, such as individuals with lower education or income, those living alone, people with disabilities, and rural residents. Targeted and accessible support for these groups may further enhance equity in digital adaptation.

Satisfaction with the daily life changes stemming from digital transformation may facilitate individuals’ openness to engaging with digital environments and, in turn, may help them realize the potential benefits of digital technologies. The active usage of digital technology provides middle-aged and older adults with access to resources and services such as information, entertainment, shopping, and banking, thereby improving their independent living, health, and life satisfaction [78-80]. The use of digital services can also predict health outcomes [78]. Based on data from 5 international longitudinal cohort studies, Li [11] found that middle-aged and older adults who use the internet reported lower levels of frailty, fewer health deficits, and better overall health. Additionally, the networks formed online can foster social connections among individuals experiencing a gradual reduction in their social roles and relationships, thereby contributing to decreased loneliness and improved well-being [19,38,81,82]. This suggests a reciprocal relationship where active digital engagement not only requires social resources but also provides opportunities to maintain or enhance them, particularly for those with limited social networks [83]. Within this framework, improving digital self-efficacy does more than facilitate technical adaptation; it may empower socially isolated individuals to manage and expand their social capital through digital means.

By examining individuals’ satisfaction with the daily life changes stemming from digital transformation, this study highlights the evolving challenges of the digital era. These challenges are particularly relevant in the post–COVID-19 period, as digital competencies have become essential for life satisfaction. South Korea provides a unique context for this study, as it is the fastest-aging society among the OECD countries [84] and is undergoing rapid digital transformation [6]. Our findings not only contribute to the literature on aging and digitalization but also provide actionable insights for addressing similar challenges worldwide.

### Strengths and Limitations

This study used nationally representative survey data from 2985 middle-aged and 1170 older adults in South Korea. The survey was conducted by reputable organizations and government agencies, including the Ministry of Science and ICT in collaboration with the National Information Society Agency. Trained interviewers visited participants’ residences to administer the questionnaires in a standardized manner. In addition, the dataset contained no missing values for the variables used in this study, eliminating the need for casewise deletion or imputation. Therefore, the large sample size and rigorous data collection methods enhance the reliability and generalizability of our findings, offering valuable insights for the older adult population.

Nevertheless, this study has several limitations. First, this study focused on the general population and did not include specific groups such as people with disabilities or those living alone, who may experience distinct challenges in digital integration. Future research should explicitly include these populations and use accessibility-focused measures, tailored digital interventions, or subgroup-specific analyses to better understand digital integration across diverse groups. Second, the instrument used to measure social capital assessed both online and offline social capital, and it was not possible to differentiate between the two in the results. Therefore, the findings reflect overall social capital rather than mode-specific resources, and future research should disentangle offline and online dimensions to examine whether their moderating roles differ. Third, the cross-sectional design precludes the establishment of a clear causal relationship between digital self-efficacy and satisfaction with the daily life changes stemming from digital transformation. It remains unclear whether higher levels of digital self-efficacy lead to greater satisfaction or vice versa. Future studies should use a longitudinal design to explore these causal relationships. Fourth, key variables were measured using self-reported questionnaires, which may be subject to recall or social desirability bias. Fifth, although we adjusted for digital competence, some conceptual overlap between digital competence and digital self-efficacy may remain. Future studies should further disentangle these constructs using more refined measures or alternative model specifications. Sixth, because this study was conducted in South Korea, which is characterized by rapid digital transformation and distinct sociocultural features, generalizability to other countries or cultural settings may be limited. Additionally, most participants had completed high school or above (approximately 80%), which may further limit generalizability to individuals with lower educational attainment. Seventh, although our study focused on digital self-efficacy in relation to digital transformation–related satisfaction, we should also consider the possibility of problematic internet use and addiction. Previous studies have also shown that spending excessive time on online activities increases the risk of depressive symptoms [85], sleep disturbance [86], and musculoskeletal pain [87] in this population. Accordingly, future research should assess not only the benefits of digital engagement but also its negative consequences, such as digital burden, fatigue, and cognitive load, to provide a more balanced understanding of its impact on well-being.

### Conclusion

The findings of this study demonstrate the important role of digital self-efficacy in improving satisfaction with the daily life changes stemming from digital transformation, particularly among older adults with low social capital. This study not only contributes to the literature on aging and digitalization but also offers practical insights for reducing the digital divide. The results emphasize the importance of fostering digital self-efficacy through strategies such as mentorship programs, observational learning, and tailored educational interventions. These strategies can empower vulnerable populations to adapt to digital transformation.

## Supplementary material

10.2196/79845 Multimedia Appendix 1 Confirmatory factor analysis results for the latent variable used in the multiple indicators multiple causes (MIMIC) model.

10.2196/79845 Multimedia Appendix 2 Goodness-of-fit indices for the multiple indicators multiple causes (MIMIC) model.

10.2196/79845 Multimedia Appendix 3 Multiple indicators multiple causes (MIMIC) model for satisfaction with daily life changes stemming from digital transformation in the total sample, middle-aged adults, and older adults.

10.2196/79845 Multimedia Appendix 4 Age-specific associations between digital self-efficacy, social capital, and satisfaction with the daily life changes stemming from digital transformation (N=4155).

10.2196/79845 Checklist 1 STROBE checklist.

## Abbreviations

AVE: average variance extracted
CFA: confirmatory factor analysis
CFI: comparative fit index
COR: conservation of resources
CR: composite reliability
ICT: information and communication technology
MIMIC: multiple indicators multiple causes
OECD: Organisation for Economic Co-operation and Development
Q-Q: quantile-quantile
RMSEA: root mean-square-error of approximation
SEM: structural equation modeling
SRMR: standardized root-mean-square residual
TLI: Tucker-Lewis index
