# Supplementary material for: Digital Self-Efficacy, Satisfaction With the Daily Life Changes Stemming From Digital Transformation, and the Moderating Effect of Social Capital in Middle-Aged and Older Adults: Cross-Sectional Survey Study
Source: JMIR Aging. 2026 Jul 31;9:e79845. doi: 10.2196/79845 (PMC13426123; doi:10.2196/79845)
Supplement: Multimedia Appendix 1 [file aging-v9-e79845-s001.docx]

Multimedia Appendix 1. Confirmatory factor analysis results for the latent variable used in the multiple indicator multiple cause (MIMIC) model

|  | Factor loading  β^a^ (*P*) | CR^b^ | AVE^c^ |
| --- | --- | --- | --- |
| Total (N=4,155) |  | 0.9 | 0.7 |
| Item 1: Leisure opportunities | 0.83 (<.001) |  |  |
| Item 2: Access to information and knowledge | 0.82 (<.001) |  |  |
| Item 3: Social connection and exchange | 0.84 (<.001) |  |  |
| Item 4: Work and study efficiency | 0.85 (<.001) |  |  |
| Middle aged adults (n=2,985) |  | 0.87 | 0.62 |
| Item 1: Leisure opportunities | 0.78 (<.001) |  |  |
| Item 2: Access to information and knowledge | 0.76 (<.001) |  |  |
| Item 3: Social connection and exchange | 0.79 (<.001) |  |  |
| Item 4: Work and study efficiency | 0.82 (<.001) |  |  |
| Older adults (n=1,170) |  | 0.92 | 0.73 |
| Item 1: Leisure opportunities | 0.84 (<.001) |  |  |
| Item 2: Access to information and knowledge | 0.85 (<.001) |  |  |
| Item 3: Social connection and exchange | 0.87 (<.001) |  |  |
| Item 4: Work and study efficiency | 0.86 (<.001) |  |  |

^a^β: standardized regression coefficient.

^b^CR: composite reliability.

^c^AVE: average variance extracted.
