# Supplementary material for: Digital Self-Efficacy, Satisfaction With the Daily Life Changes Stemming From Digital Transformation, and the Moderating Effect of Social Capital in Middle-Aged and Older Adults: Cross-Sectional Survey Study
Source: JMIR Aging. 2026 Jul 31;9:e79845. doi: 10.2196/79845 (PMC13426123; doi:10.2196/79845)
Supplement: Multimedia Appendix 2 [file aging-v9-e79845-s002.docx]

Multimedia Appendix 2. Goodness-of-fit indices for the multiple indicator multiple cause (MIMIC) model

|  | Goodness-of-fit indicators | | |
| --- | --- | --- | --- |
|  | CFI^a^ | TLI^b^ | SRMR^c^ |
| Total (N=4,155) | 0.99 | 0.99 | 0.01 |
| Middle aged adults (n=2,985) | 0.99 | 0.99 | 0.01 |
| Older adults (n=1,170) | 0.99 | 0.99 | 0.01 |

^a^CFI: comparative fit index.

^b^TLI: Tucker–Lewis index.

^c^SRMR: standardized root mean square residual.
