# Supplementary material for: Digital Self-Efficacy, Satisfaction With the Daily Life Changes Stemming From Digital Transformation, and the Moderating Effect of Social Capital in Middle-Aged and Older Adults: Cross-Sectional Survey Study
Source: JMIR Aging. 2026 Jul 31;9:e79845. doi: 10.2196/79845 (PMC13426123; doi:10.2196/79845)
Supplement: Multimedia Appendix 3 [file aging-v9-e79845-s003.docx]

Multimedia Appendix 3. Multiple indicators multiple causes (MIMIC) model for satisfaction with daily life changes stemming from digital transformation in the total sample, middle-aged adults, and older adults

| Path | | β^a^ | SE^b^ | *P* | 95% CI^c^ |
| --- | --- | --- | --- | --- | --- |
| Total (N=4,155) | | | | |  |
|  | H1. Digital self-efficacy → Satisfaction^d^ | 0.47 | 0.02 | <.001 | [0.43, 0.50] |
|  | H2. Social capital → Satisfaction | 0.12 | 0.02 | <.001 | [0.09, 0.15] |
|  | H3. Digital self-efficacy × Social capital → Satisfaction | −0.05 | 0.01 | <.001 | [−0.08, −0.02] |
|  | H4. Age → Satisfaction | −0.04 | 0.02 | .03 | [−0.07, −0.01] |
|  | H5. Gender → Satisfaction | 0.01 | 0.01 | .31 | [−0.01, 0.04] |
|  | H6. Education level → Satisfaction | 0.07 | 0.02 | <.001 | [0.03, 0.10] |
|  | H7. Living arrangement → Satisfaction | <0.001 | 0.01 | .97 | [−0.03, 0.03] |
|  | H8. Presence of disability → Satisfaction | −0.003 | 0.01 | .78 | [−0.03, 0.02] |
|  | H9. Monthly household income → Satisfaction | 0.05 | 0.02 | <.001 | [0.02, 0.08] |
|  | H10. Self-rated health → Satisfaction | −0.02 | 0.01 | .12 | [−0.05, 0.01] |
|  | H11. Digital competence → Satisfaction | 0.17 | 0.02 | <.001 | [0.13, 0.20] |
| Middle-aged adults (n=2,985) | | | | |  |
|  | H1. Digital self-efficacy → Satisfaction | 0.46 | 0.02 | <.001 | [0.42, 0.50] |
|  | H2. Social capital → Satisfaction | 0.18 | 0.02 | <.001 | [0.14, 0.22] |
|  | H3. Digital self-efficacy × Social capital → Satisfaction | −0.05 | 0.02 | .01 | [−0.09, −0.01] |
|  | H4. Age → Satisfaction | −0.02 | 0.02 | .34 | [−0.05, 0.02] |
|  | H5. Gender → Satisfaction | 0.02 | 0.02 | .32 | [−0.02, 0.05] |
|  | H6. Education level → Satisfaction | 0.04 | 0.02 | .03 | [0.01, 0.08] |
|  | H7. Living arrangement → Satisfaction | −0.001 | 0.02 | .94 | [−0.03, 0.03] |
|  | H8. Presence of disability → Satisfaction | −0.02 | 0.01 | .15 | [−0.05, 0.01] |
|  | H9. Monthly household income → Satisfaction | 0.07 | 0.02 | <.001 | [0.04, 0.11] |
|  | H10. Self-rated health → Satisfaction | −0.03 | 0.02 | .11 | [−0.06, 0.01] |
|  | H11. Digital competence → Satisfaction | 0.13 | 0.02 | <.001 | [0.09, 0.17] |
| Older adults (n=1,170) | | | | |  |
|  | H1. Digital self-efficacy → Satisfaction | 0.44 | 0.03 | <.001 | [0.38, 0.51] |
|  | H2. Social capital → Satisfaction | −0.01 | 0.04 | .89 | [−0.09, 0.08] |
|  | H3. Digital self-efficacy × Social capital → Satisfaction | −0.15 | 0.04 | <.001 | [−0.23, −0.07] |
|  | H4. Age → Satisfaction | −0.04 | 0.03 | .08 | [−0.09, 0.01] |
|  | H5. Gender → Satisfaction | −0.01 | 0.02 | .74 | [−0.06, 0.04] |
|  | H6. Education level → Satisfaction | 0.06 | 0.03 | .04 | [0.002, 0.11] |
|  | H7. Living arrangement → Satisfaction | −0.02 | 0.03 | .41 | [−0.07, 0.03] |
|  | H8. Presence of disability → Satisfaction | 0.03 | 0.02 | .22 | [−0.02, 0.07] |
|  | H9. Monthly household income → Satisfaction | 0.01 | 0.03 | .68 | [−0.04, 0.06] |
|  | H10. Self-rated health → Satisfaction | −0.02 | 0.03 | .37 | [−0.08, 0.03] |
|  | H11. Digital competence → Satisfaction | 0.2 | 0.03 | <.001 | [0.14, 0.26] |

^a^β: standardized regression coefficient.

^b^SE: standard error.

^c^CI: confidence interval.

^d^Satisfaction: Satisfaction with the daily life changes stemming from digital transformation.
