# Supplementary material for: Digital Self-Efficacy, Satisfaction With the Daily Life Changes Stemming From Digital Transformation, and the Moderating Effect of Social Capital in Middle-Aged and Older Adults: Cross-Sectional Survey Study
Source: JMIR Aging. 2026 Jul 31;9:e79845. doi: 10.2196/79845 (PMC13426123; doi:10.2196/79845)
Supplement: Multimedia Appendix 4 [file aging-v9-e79845-s004.docx]

Multimedia Appendix 4. Age-specific associations between digital self-efficacy, social capital, and satisfaction with the daily life changes stemming from digital transformation (N=4,155)

|  | Model 1 | | Model 2 | | Model 3 | | Model 4 | |
| --- | --- | --- | --- | --- | --- | --- | --- | --- |
|  | 40−50 years  (n=1,189) | | 51−64 years  (n=1,796) | | 65−74 years  (n=872) | | 75 years and older  (n=298) | |
| Variable | B^a^ (SE)^b^ | *t* (*P*) | B (SE) | *t* (*P*) | B (SE) | *t* (*P*) | B (SE) | *t* (*P*) |
| **Independent variable** |  |  |  |  |  |  |  |  |
| Digital self-efficacy | 0.52 (0.04) | 14.58 (<.001) | 0.49 (0.03) | 17.35 (<.001) | 0.60 (0.04) | 14.71 (<.001) | 0.54 (0.08) | 6.52 (<.001) |
| **Moderation variable** |  |  |  |  |  |  |  |  |
| Social capital | 0.32 (0.05) | 6.76 (<.001) | 0.25 (0.04) | 6.13 (<.001) | 0.11 (0.05) | 2.06 (.04) | 0.12 (0.10) | 1.22 (.22) |
| **Interaction term** |  |  |  |  |  |  |  |  |
| Digital self-efficacy × Social capital | −0.15 (0.07) | −2.24 (.03) | −0.14 (0.05) | −2.88 (.004) | −0.27 (0.07) | −3.88 (<.001) | −0.21 (0.16) | −1.34 (.18) |
| **Control variables**^c^ |  |  |  |  |  |  |  |  |
| Age | −0.003 (0.005) | −0.53 (.59) | −0.002 (0.004) | −0.52 (.60) | −0.02 (0.01) | −2.11 (.04) | −0.02 (0.01) | −1.59 (.11) |
| Gender (ref ^d^: male) | 0.01 (0.03) | 0.32 (.75) | 0.03 (0.03) | 1.05 (.30) | −0.01 (0.05) | −0.11 (.91) | −0.04 (0.09) | −0.43 (.67) |
| Education level (ref: Middle school or lower) | 0.22 (0.16) | 1.38 (.17) | 0.11 (0.06) | 2.00 (.05) | 0.07 (0.05) | 1.34 (.18) | 0.17 (0.11) | 1.49 (.14) |
| Living arrangements (ref: living alone) | −0.11 (0.07) | −1.51 (.13) | 0.05 (0.06) | 0.84 (.40) | −0.08 (0.07) | −1.13 (.26) | −0.01 (0.10) | −0.06 (.95) |
| Presence of disability (ref: without disability) | 0.04 (0.11) | 0.37 (.71) | −0.16 (0.09) | −1.70 (.09) | 0.31 (0.18) | 1.69 (.09) | −0.06 (0.22) | −0.26 (.80) |
| Monthly household income (ref: < 4,000,000^e^) | 0.17 (0.04) | 4.03 (<.001) | 0.07 (0.03) | 2.21 (.03) | 0.01 (0.06) | 0.18 (.86) | 0.13 (0.16) | 0.82 (.41) |
| Self-rated health | 0.003 (0.03) | 0.10 (.92) | −0.05 (0.03) | −2.01 (.05) | −0.06 (0.04) | −1.61 (.11) | 0.04 (0.06) | 0.62 (.53) |
| Digital competence | 0.04 (0.02) | 2.31 (.02) | 0.08 (0.01) | 6.44 (<.001) | 0.13 (0.02) | 6.49 (<.001) | 0.12 (0.04) | 2.89 (.004) |

^a^B: unstandardized regression coefficients.

^b^SE: standard error.

^c^All models were adjusted for the same set of covariates (age, gender, education level, living arrangements, disability, household income, self-rated health, and digital competence).

^d^ref: reference category.

^e^A currency exchange rate of KRW 4,000,000=US $ 3,096.81 (KRW 1=US $ 0.000774; 2022 annual average, World Bank) was applied [63].
